# Histomorphological and Immunohistochemical Profile of Malignant Breast Lesions in a Tertiary Care Center of Central India: A Cross-Sectional Study

**DOI:** 10.7759/cureus.114826

**Published:** 2026-08-20

**Authors:** Himani Himani, Atul Jain, Shikha Agarwal, Amar Gangwani

**Affiliations:** 1 Pathology, Bundelkhand Medical College, Sagar, IND; 2 Pathology and Laboratory Medicine, Bundelkhand Medical College, Sagar, IND

**Keywords:** breast carcinoma, estrogen receptor, her2/neu, histopathology, immunohistochemistry

## Abstract

Introduction: Breast carcinoma is a heterogeneous malignancy with variable histomorphological patterns and immunohistochemical profiles. Evaluation of estrogen receptor (ER), progesterone receptor (PR), and human epidermal growth factor receptor 2 (HER2/neu) expression provides important prognostic and predictive information for treatment planning. Ki-67 was not evaluated in the present study; therefore, the reported immunohistochemical subgroups were based only on ER, PR, and HER2/neu expression. This study aimed to assess the histomorphological and immunohistochemical profile of malignant breast lesions in a tertiary care center in Central India.

Methods: This cross-sectional observational study included 84 patients with histopathologically confirmed malignant breast lesions. Tumors were classified according to standard histopathological criteria and graded using the Nottingham modification of the Bloom-Richardson system. Immunohistochemistry was performed for ER, PR, and HER2/neu. Molecular subtypes were assigned based on ER, PR, and HER2/neu expression.

Results: The most common age group was 41-50 years (n = 39, 46.4%). Invasive ductal carcinoma, not otherwise specified, accounted for 95.2% (80/84) of cases. Grade III tumors were observed in 58.3% (49/84), and lymph node metastasis was present in 58.3% (49/84). ER, PR, and HER2/neu positivity were observed in 59.5%, 53.6%, and 29.8% of cases, respectively. The hormone receptor (HR)-positive/HER2-negative subgroup was the most common (59.5%), followed by HR-positive/HER2-positive (17.9%), HR-negative/HER2-positive (11.9%), and triple-negative (10.7%) subgroups. On descriptive analysis, HR positivity decreased with increasing tumor grade and nodal positivity, whereas HER2/neu positivity increased.

Conclusion: Histopathological evaluation combined with ER, PR, and HER2/neu immunohistochemistry provides important information for tumor classification, prognostic assessment, and therapeutic decision-making in breast carcinoma. Routine integrated reporting is essential for individualized management of malignant breast lesions.

## Introduction

Breast carcinoma is a biologically heterogeneous disease and remains a major public health problem worldwide. In 2022, female breast cancer accounted for approximately 2.3 million new cases globally, and it continues to be among the most common causes of cancer morbidity and mortality in women [1]. In India, the burden is increasing, with breast cancer now representing one of the leading cancers among women [2,3].

Delayed presentation, limited screening, and regional differences in health-seeking behavior influence the tumor stage and biological profile at diagnosis [2,3].

Routine histopathological assessment remains the foundation of diagnosis. The World Health Organization classification provides standardized criteria for categorizing breast tumors, while histological grading using the Nottingham modification of the Bloom-Richardson system provides important prognostic information by assessing tubule formation, nuclear pleomorphism, and mitotic activity [4,5]. However, morphology alone does not fully explain the clinical behavior of breast carcinoma.

Immunohistochemistry has therefore become an essential component of routine breast cancer evaluation. Estrogen receptor (ER) and progesterone receptor (PR) status predict response to endocrine therapy, whereas human epidermal growth factor receptor 2 (HER2) overexpression identifies patients who may benefit from HER2-targeted therapy [6,7]. Immunohistochemical surrogates also enable practical classification into Luminal A, Luminal B, HER2-enriched, and triple-negative subtypes, which differ in prognosis, recurrence patterns, and therapeutic responses [8-10].

As clinicopathological patterns and receptor distribution may vary across populations, region-specific data are clinically relevant. The present study was conducted to evaluate the histomorphological spectrum and immunohistochemical profile of malignant breast lesions in Central India and to assess their association with clinicopathological parameters.

## Materials and methods

Study design and setting

This was a cross-sectional observational study conducted in the Department of Pathology, Bundelkhand Medical College, Sagar, Madhya Pradesh, India, for 18 months from May 2024 to December 2025. The study included patients with histopathologically confirmed malignant breast lesions who underwent biopsy or surgical resection during the study period.

Study population

A total of 84 cases of malignant breast lesions were included in the study. All cases diagnosed as malignant epithelial breast tumors on histopathological examination were considered eligible for inclusion. Clinical details, including age, menopausal status, lesion laterality, specimen type, and lymph node status, were recorded from requisition forms, clinical case records, and histopathology records.

Ethical considerations

The study was conducted after obtaining approval from the Institutional Ethics Committee of Bundelkhand Medical College, Sagar, Madhya Pradesh, India (IECBMC/DHR/2024/59, dated March 21, 2024). Written informed consent was waived for the study. Confidentiality of patient identity and clinical information was maintained throughout the study.

Inclusion criteria

All female patients with histopathologically confirmed malignant breast lesions were included. Both biopsy and surgical resection specimens were considered eligible, provided that adequate tissue was available for routine histopathological evaluation and immunohistochemical analysis.

Exclusion criteria

Cases with benign breast lesions, recurrent breast carcinoma, male breast carcinoma, inadequate tissue for immunohistochemistry, poorly preserved specimens, or a history of neoadjuvant chemotherapy or radiotherapy before tissue diagnosis were excluded.

Histopathological evaluation

All specimens were fixed in 10% neutral buffered formalin and processed routinely. Paraffin-embedded tissue sections of 3-5 μm thickness were prepared and stained with hematoxylin and eosin. Histological typing was performed according to the World Health Organization classification of breast tumors [4]. Tumors were graded using the Nottingham modification of the Bloom-Richardson grading system, which assesses tubule formation, nuclear pleomorphism, and mitotic count [5]. Based on the total score, tumors were categorized as Grade I, Grade II, or Grade III.

Lymph node status was assessed in modified radical mastectomy specimens whenever axillary lymph nodes were available. Cases were categorized as node-positive or node-negative based on the presence or absence of metastatic tumor deposits in examined lymph nodes.

Immunohistochemical analysis

Immunohistochemistry was performed on formalin-fixed, paraffin-embedded tissue sections using antibodies against ER, PR, and HER2/neu. Sections were mounted on poly-L-lysine-coated slides, deparaffinized, rehydrated, and subjected to antigen retrieval. After blocking endogenous peroxidase activity, sections were incubated with primary antibodies and then with a polymer-based detection system. Diaminobenzidine was used as the chromogen, and hematoxylin was used for counterstaining.

ER and PR staining were interpreted according to the American Society of Clinical Oncology/College of American Pathologists (ASCO/CAP) guideline recommendations. Nuclear staining in ≥1% of invasive tumor cells was considered positive for ER or PR [6]. HER2/neu expression was evaluated according to ASCO/CAP recommendations and scored as 0, 1+, 2+, or 3+ based on the intensity and completeness of membranous staining in invasive tumor cells [7]. For analysis, cases with an HER2 score of 3+ were considered positive, while scores of 0 and 1+ were considered negative. Equivocal HER2 cases with a score of 2+ were considered according to the available institutional protocol; if confirmatory testing was not available, they were not categorized as HER2-positive.

Molecular subtyping

Based on ER, PR, and HER2 immunohistochemical expression, cases were categorized into surrogate molecular subtypes. Tumors positive for ER and/or PR and negative for HER2 were classified as Luminal A-like. Tumors positive for ER and/or PR and HER2-positive were classified as Luminal B-like. Tumors negative for ER and PR but positive for HER2 were classified as HER2-enriched. Tumors negative for ER, PR, and HER2 were classified as triple-negative breast carcinoma. This immunohistochemistry-based classification was used as a practical surrogate for molecular classification in routine diagnostic pathology [8,10].

Data collection

Data were entered in a predesigned structured proforma. The recorded variables included age group, menopausal status, laterality, histological type, tumor grade, lymph node status, ER status, PR status, HER2 status, and molecular subtype. All categorical data were expressed as frequencies and percentages.

Statistical analysis

Data were compiled and analyzed descriptively. All categorical variables, including age group, menopausal status, laterality, histological type, tumor grade, lymph node status, ER status, PR status, HER2/neu status, and molecular subtype, were expressed as frequencies and percentages. No inferential statistical tests were applied, and no p-values were calculated. Therefore, the observed differences in receptor expression by tumor grade and lymph node status were interpreted descriptively as trends rather than statistically tested for associations.

## Results

Demographic and clinical characteristics

The most common age group was 41-50 years, comprising 39 patients (46.4%), followed by >60 years with 20 patients (23.8%), 31-40 years with 15 patients (17.9%), and 51-60 years with 10 patients (11.9%). With respect to menopausal status, 32 patients (38.1%) were premenopausal, 20 (23.8%) were perimenopausal, and 32 (38.1%) were postmenopausal. Most cases were unilateral, observed in 80 patients (95.2%), while bilateral involvement was noted in four patients (4.8%). Lymph node metastasis was present in 49 patients (58.3%), whereas 35 patients (41.7%) were node-negative (Table 1).

**Table 1:** Demographic and clinical characteristics of the study population

Variable	Category	Frequency (n = 84)	Percentage (%)
Age group (years)	31-40	15	17.9
	41-50	39	46.4
	51-60	10	11.9
	>60	20	23.8
Menopausal status	Premenopausal	32	38.1
	Perimenopausal	20	23.8
	Postmenopausal	32	38.1
Laterality	Unilateral	80	95.2
	Bilateral	4	4.8
Lymph node status	Positive	49	58.3
	Negative	35	41.7

Histopathological characteristics

Invasive ductal carcinoma, not otherwise specified (NOS), was the most common histological type (n = 80, 95.2%). Grade III tumors predominated (n = 49, 58.3%) (Table 2).

**Table 2:** Histopathological characteristics of malignant breast lesions NOS: Not otherwise specified.

Parameter	Category	Frequency (n = 84)	Percentage (%)
Histological type	Invasive ductal carcinoma, NOS	80	95.2
	Invasive ductal carcinoma with medullary features	4	4.8
Tumor grade	Grade I	15	17.9
	Grade II	20	23.8
	Grade III	49	58.3

Immunohistochemical profile

On immunohistochemical evaluation, ER positivity was observed in 50 patients (59.5%), while 34 patients (40.5%) were ER-negative. Progesterone receptor positivity was found in 45 patients (53.6%), and 39 patients (46.4%) were PR-negative. HER2/neu positivity was observed in 25 patients (29.8%), while 59 patients (70.2%) were HER2-negative (Table 3).

**Table 3:** Immunohistochemical profile of malignant breast lesions ER: Estrogen receptor; PR: Progesterone receptor; HER2/neu: Human epidermal growth factor receptor 2/neu oncogene; n: Number of cases; %: Percentage.

Marker	Positive, n (%)	Negative, n (%)
ER	50 (59.5)	34 (40.5)
PR	45 (53.6)	39 (46.4)
HER2/neu	25 (29.8)	59 (70.2)

Molecular subtype distribution

Based on ER, PR, and HER2/neu expression, the most common molecular subtype was Luminal A, identified in 50 patients (59.5%). Luminal B subtype was observed in 15 patients (17.9%), HER2-enriched subtype in 10 patients (11.9%), and triple-negative breast carcinoma in nine patients (10.7%) (Table 4).

**Table 4:** Distribution of molecular subtypes

Molecular subtype	Frequency (n = 84)	Percentage (%)
Luminal A	50	59.5
Luminal B	15	17.9
HER2-enriched	10	11.9
Triple-negative breast carcinoma	9	10.7

Association of tumor grade with receptor status

Hormone receptor (HR) expression showed a decreasing trend as tumor grade increased. ER positivity was present in 15 of 15 Grade I tumors (100.0%), 20 of 20 Grade II tumors (100.0%), and 15 of 49 Grade III tumors (30.6%). Similarly, PR positivity was observed in 14 of 15 Grade I tumors (93.3%), 16 of 20 Grade II tumors (80.0%), and 15 of 49 Grade III tumors (30.6%). In contrast, HER2/neu positivity increased with tumor grade, being present in 1 of 15 Grade I tumors (6.7%), 5 of 20 Grade II tumors (25.0%), and 19 of 49 Grade III tumors (38.8%) (Table 5).

**Table 5:** Association of tumor grade with receptor status

Tumor grade	ER-positive, n (%)	PR-positive, n (%)	HER2-positive, n (%)
Grade I (n = 15)	15 (100.0)	14 (93.3)	1 (6.7)
Grade II (n = 20)	20 (100.0)	16 (80.0)	5 (25.0)
Grade III (n = 49)	15 (30.6)	15 (30.6)	19 (38.8)

Association of lymph node status with receptor status

Among node-negative cases, ER positivity was observed in 28 of 35 patients (80.0%), PR positivity in 26 of 35 patients (74.3%), and HER2/neu positivity in 5 of 35 patients (14.3%). Among node-positive cases, ER positivity was observed in 22 of 49 patients (44.9%), PR positivity in 19 of 49 patients (38.8%), and HER2/neu positivity in 20 of 49 patients (40.8%).

HR positivity was more frequent among node-negative tumors, whereas HER2/neu positivity was more common among node-positive tumors. This pattern suggests that ER/PR negativity and HER2/neu positivity were associated with more aggressive disease characteristics, including lymph node metastasis (Table 6).

**Table 6:** Association of lymph node status with receptor expression

Lymph node status	ER-positive, n (%)	PR-positive, n (%)	HER2-positive, n (%)
Node-negative (n = 35)	28 (80.0)	26 (74.3)	5 (14.3)
Node-positive (n = 49)	22 (44.9)	19 (38.8)	20 (40.8)

## Discussion

The present study evaluated the histomorphological and immunohistochemical profiles of malignant breast lesions at a tertiary care center in Central India. A total of 84 patients were included. The major findings were predominance of invasive ductal carcinoma, NOS, a high proportion of Grade III tumors, frequent lymph node positivity, and clinically relevant variation in ER, PR, and HER2/neu expression according to tumor grade and nodal status. These findings support the importance of combining routine histopathology with immunohistochemistry for accurate classification, prognostic assessment, and therapeutic planning in breast carcinoma [1,4,6,7].

Breast carcinoma remains one of the most common malignancies among women worldwide. The Global Cancer Observatory (GLOBOCAN) 2022 reported breast cancer as a major contributor to global cancer incidence and cancer-related mortality among women [1]. In India, the burden of breast cancer has continued to increase, and population-based cancer registry data have shown that breast cancer is one of the leading cancers among Indian women [2]. The need for region-specific studies is evident because age at diagnosis, stage at presentation, histological grade, and receptor expression may vary across regions due to differences in awareness, screening practices, socioeconomic factors, and access to healthcare [2,3].

In the present study, the most common age group was 41-50 years, comprising 39 patients (46.4%). This finding suggests that many patients presented during the perimenopausal age group. Malvia et al. reported that breast cancer among Indian women is commonly seen at a relatively younger age compared with Western populations [3]. Chand et al. also reported a similar trend in an Indian cohort evaluating the immunohistochemical profile of breast cancer [11]. Younger age at presentation is clinically important because it may be associated with higher-grade tumors, delayed diagnosis, and biologically aggressive disease patterns.

Invasive ductal carcinoma, NOS, was the predominant histological type in the present study and was observed in 80 patients (95.2%). Invasive ductal carcinoma with medullary features was seen in four patients (4.8%). This predominance is consistent with the World Health Organization classification, where invasive breast carcinoma of no special type forms the largest category of invasive breast cancers [4]. Similar observations have been reported in clinicopathological studies from India, where invasive ductal carcinoma remains the most frequent histological type [11,12]. Histological typing remains relevant because certain special subtypes may show distinct biological behavior and prognosis.

A major finding in this study was the high proportion of Grade III tumors. Grade III tumors were observed in 49 patients (58.3%), followed by Grade II tumors in 20 patients (23.8%) and Grade I tumors in 15 patients (17.9%). Tumor grading using the Nottingham modification of the Bloom-Richardson system is a well-established prognostic tool in invasive breast carcinoma [5]. Rakha et al. demonstrated that Nottingham histological grade has independent prognostic significance and remains useful even in the molecular era [6]. The predominance of high-grade tumors in the present study may reflect delayed presentation, lack of organized screening, and advanced biological behavior at the time of diagnosis.

Lymph node metastasis was present in 49 patients (58.3%), while 35 patients (41.7%) were node-negative. Axillary lymph node status is one of the most important prognostic factors in breast carcinoma and is closely related to staging, recurrence risk, and the need for adjuvant therapy. The high proportion of node-positive cases in the present study suggests that a significant number of patients presented with locally or regionally advanced disease. Similar associations between nodal status and adverse tumor biology have been described in studies evaluating receptor expression and clinicopathological parameters [12,13].

In the present study, ER positivity was observed in 50 patients (59.5%), and PR positivity in 45 patients (53.6%). HR testing is recommended for all invasive breast carcinomas because ER and PR expression provide predictive information for endocrine therapy and prognostic information for disease behavior [7]. The HR positivity observed in this study indicates that more than half of the patients may benefit from endocrine therapy. However, the proportion of HR-positive cases appears to be lower than that reported in many Western cohorts, possibly due to a younger age at presentation, higher tumor grade, and delayed diagnosis in the Indian setting [3,11].

HER2/neu positivity was observed in 25 patients (29.8%), while 59 patients (70.2%) were HER2-negative. HER2 testing is an essential component of breast cancer workup because HER2-positive tumors are biologically aggressive but may respond to HER2-targeted therapy [8]. The HER2 positivity rate in the present study falls within the broad range reported in the literature. Park et al. also showed that immunohistochemistry-based subtyping using ER, PR, HER2, and Ki-67 provides clinically useful information regarding tumor biology and outcomes [14].

Based on ER, PR, and HER2/neu expression, Luminal A was the most common subtype, observed in 50 patients (59.5%). Luminal B was seen in 15 patients (17.9%), HER2-enriched subtype in 10 patients (11.9%), and triple-negative breast carcinoma in nine patients (10.7%). Molecular classification of breast cancer was first established through gene-expression profiling by Perou et al., who demonstrated that breast cancers represent biologically distinct molecular entities [8]. In routine practice, immunohistochemistry-based surrogate classification is widely used because gene-expression profiling is not always feasible, particularly in resource-limited settings [10,14]. The predominance of Luminal A in the present study indicates that HR-positive tumors formed the largest subgroup. However, identification of HER2-enriched and triple-negative tumors remains important because these subtypes usually require different therapeutic strategies and closer clinical monitoring [14,15].

The association between tumor grade and receptor expression was clinically meaningful. ER positivity was observed in 15 of 15 Grade I tumors (100.0%), 20 of 20 Grade II tumors (100.0%), and 15 of 49 Grade III tumors (30.6%). Similarly, PR positivity decreased from 14 of 15 Grade I tumors (93.3%) and 16 of 20 Grade II tumors (80.0%) to 15 of 49 Grade III tumors (30.6%). This inverse relationship between histological grade and HR expression is biologically plausible because well-differentiated tumors are more likely to retain HR expression. In contrast, poorly differentiated tumors are more frequently HR-negative [6,7,12,13].

In contrast, HER2/neu positivity increased with tumor grade. HER2/neu positivity was observed in 1 of 15 Grade I tumors (6.7%), 5 of 20 Grade II tumors (25.0%), and 19 of 49 Grade III tumors (38.8%). This pattern suggests that HER2/neu overexpression was more common among poorly differentiated tumors. Azizun-Nisa et al. reported that HER2/neu expression was associated with higher tumor grade, larger tumor size, and lymph node involvement [13]. This supports the present finding that HER2/neu positivity is linked with aggressive clinicopathological features.

The relationship between lymph node status and receptor expression also showed a clear pattern. Among node-negative cases, ER positivity was seen in 28 of 35 patients (80.0%), PR positivity in 26 of 35 patients (74.3%), and HER2/neu positivity in 5 of 35 patients (14.3%). Among node-positive cases, ER positivity was observed in 22 of 49 patients (44.9%), PR positivity in 19 of 49 patients (38.8%), and HER2/neu positivity in 20 of 49 patients (40.8%). These findings indicate that HR-positive tumors were more frequent in node-negative disease, while HER2/neu positivity was more frequent among node-positive tumors. Gupta et al. similarly reported correlations between HR expression and histological parameters in breast tumors [12]. These observations suggest that receptor status should be interpreted together with tumor grade and nodal status rather than in isolation.

Triple-negative breast carcinoma was observed in nine patients (10.7%) in the present study. Although this subgroup was smaller than the luminal group, its identification is important because triple-negative breast cancer is associated with aggressive clinical behavior, limited endocrine or HER2-targeted treatment options, and distinct recurrence patterns. Dent et al. reported that triple-negative breast cancer has characteristic clinical features and recurrence behavior compared with other breast cancer subtypes [15]. Therefore, even a relatively small proportion of triple-negative cases has important clinical implications.

Overall, the present study reinforces the value of integrated histopathological and immunohistochemical evaluation in malignant breast lesions. Histological type, tumor grade, lymph node status, ER, PR, HER2/neu expression, and molecular subtype together provide a more complete assessment of tumor biology. In tertiary care and resource-limited settings, immunohistochemistry remains a practical and cost-effective method for guiding decisions on endocrine therapy, HER2-targeted therapy, chemotherapy, and prognostic stratification [7,8,10,14].

The present study has certain limitations. It was a single-center study with a sample size of 84 patients, which may limit the generalizability of the findings. The cross-sectional design did not allow assessment of treatment response, recurrence, disease-free survival, or overall survival. Ki-67 was not included in the immunohistochemical panel, which may limit further refinement of luminal subtyping. Despite these limitations, the study provides useful regional data from Central India and highlights the importance of routine ER, PR, and HER2/neu testing in all malignant breast lesions.

## Conclusions

The present study highlights the importance of combining routine histopathological evaluation with immunohistochemical profiling for assessing malignant breast lesions. Histological type, tumor grade, lymph node status, and ER, PR, and HER2 expression together provide valuable information for understanding tumor biology and guiding treatment decisions. The observed association of HR loss and HER2 expression with aggressive clinicopathological features emphasizes the need for standardized receptor testing in all invasive breast carcinomas. Integrated reporting of morphology and immunohistochemistry is essential for accurate classification, prognostic stratification, and selection of appropriate endocrine, targeted, and systemic therapy.
